# Case of Typhoid Fever, with Autopsy and Remarks

**Published:** 1845-12-01

**Authors:** G. N. Burwell


### Case of Typhoid Fever, with Autopsy, and Remarks.

Communicated for the Buffalo Medical Journal, by G. N. BURWELL, M. D.

BUFFALO, November 12, 1845.

MY DEAR DOCTOR :—In the introduction to my article on the fever at North Boston, of which you gave an account lately in the American Jour. of Med. Science, you said I promised you an account of cases which had come under my notice in this city. You misunderstood me somewhat. The case I promised was one which came under my observation in the Philadelphia Hospital, Blockley, and is interesting, pathologically, as illustrating the immense amount of disease which may exist in the intestinal canal, with comparatively slight constitutional symptoms, until a short time before death.

Before transcribing this case from my note book, I would say that from April, 1844, to April, 1845, I saw six cases in this city of what I considered as the typhoid fever of Louis, Chomel, &c. All but one were attacked in the city; all were young men—three had the eruption well marked, two did not have it, and in one it is not recollected; one died at the commencement of the third week, from the supervention of pleuro-pneumonia; the other five recovered.

Beside these, I saw a woman in December last, on Grand Island, whom I considered as having this fever. In June I went to Hamburgh, near Boston, to see a woman who undoubtedly had this fever; and in April last, my father went to the same town to see a case of this disease.

In thus citing these cases as typhoid fever, I am aware I have no farther evidence to offer respecting them than my own opinion, but which is very decided. This is one reason why I refer to them so briefly, and, also, because I neither wish nor ask others to receive them as such on this ground alone. I only desire to draw their attention to the subject, and to ask that they investigate for themselves.

I would here fortify my opinion respecting this fever, by that of Doct. J. Dorland, of Hamburgh, which he communicated to me in a letter, and from which I take the liberty of making the following extracts:

"I saw the cases at N. Boston, mentioned by Dr. Flint,and believe his views to be correct. I had a case at the same time, some two miles from Boston; a young man who got sick at Buffalo while engaged in the printing business. About the fifth day of the disease he was brought home, and soon afterwards I saw him. The late Dr. Camp was soon called in counsel, at my request; at the same time I visited with him his patients at N. Boston. Our conclusion was, that it was one disease affecting his patients and my patient.

This is a fever I have hardly been aware of in the country until within a few years, and I always dread its appearance. Diarrhœa is a very prominent symptom, with excessive debility, and frequently hæmorrhage, from some part. There is also always more or less headache and delirium in severe cases," &c.

The treatment which Dr. D. has found most beneficial in the cases which have come under his notice, has been the abstraction of about ten ounces of blood within the first five days, followed by full evacuations from the bowels by calomel,succeeded by rhubarb. After this, he is very sparing in the use of calomel, but gives a little rhubarb every second or third day. After thus relieving somewhat the congestion of the brain, he gives grain doses of ipecacuanha frequently repeated, for the bowels, adding opium when needed, either in the form of Dover's powder, or uncombined. He objects to morphine in this fever, as, according to his observation, it has a sinking, stupefying effect, without leaving the system benefited. He thinks morphine is too much relied upon in low fevers. After free depletion by the lancet, bowels, &c., he likes the use of quinine, (freely in many cases,) the acids, ipecac, and opium; their doses to be varied, of course, according to the indications.

The case I propose to give, is that of a young man, Thomas Daggert, aged twenty-one years, a wheelwright. He left his native state, (Maine) in 1843, to go to the south, yankee-like, to seek his fortune. In November of that year, while at Mobile, he had an attack of "bilious fever," from which he had a full recovery. Not succeeding in business, homesick, and without money or friends, he determined to go home, and got a passage given him on a sloop coming to Philadelphia. Here he endeavored to earn something to help him along, by hiring out to attend one of the stands which so abound on the sidewalks of that city. While thus engaged, about the first of February, 1844, he was taken with a light chill, which was followed by fever, but no sweating. The chill returned every afternoon, and lasted generally from half an hour to an hour; the fever continued all night, with thirst, and general restlessness.

For all this sickness he attended to his stand *some portion* of each day for ten or twelve days; of course the principal part of the time was spent in the house, *but not in bed*. The truth is, I expect, that he exerted all his strength and resolution to keep about for fear of losing his place, and if lost, not knowing, sick and destitute as he was, where in that great city he could lay his head. Finally he had to give up and go to bed; and on the afternoon of February 12, 1844, he was brought to the Hospital.

He had then a pulse of 120, a warm dry skin, a sense of nausea, no appetite, bowels generally constipated, a little cough, some headache, a dull flush on his cheeks, and the general expression of his face was dull and melancholy. He had had a chill about 2 o'clock.

Feb'y 13. His pulse this morning 104, quick, moderately full and large, general feverishness, tongue dry and rough in the centre, the fur on it forked, edges somewhat red, has a bitter taste in his mouth, has vomited twice of bile, bowels open twice in last fifteen hours, stools thin, has a sense of swelling of abdomen,but which is slightly retracted, tenderness on pressure in the epigastric and both hypochondriac regions, percussion resonant, complains of pain in precordial region, which is not continued, but alternates with pains in his toes and in the left arm; no cough this morning, respiration rather hurried, urine small in quantity and high colored, no headache, no eruption.

Feb'y 14. Much the same as yesterday, no sweating, increase of restlessness, more thirst, complains of less pain in abdomen on pressure, but little sleep.

Feb'y 15. Pulse risen to 130, continued fever, no return of chills since the day of his entrance, some deafness and ringing in the ears coming on, but little headache, sordes on teeth, tongue rough and dry, bowels open twice last night, stools scanty and watery, abdomen continues retracted and tympanitic, the pain in it has concentrated about the umbilicus which is exceedingly sensitive to pressure.

Feb'y 16. Pulse 140, feeble and quick, face becoming blue, sordes, tongue swollen, dry and sore, lies with mouth open, disposition to pick his nose, and some epistaxis in consequence, brain symptoms continue, delirium last night and this morning.

P. M. Increased delirium with some stupor, countenance very dull but becomes anxious when roused, belly now slightly swollen, pulse 150 small and compressible, no chills, nor eruption.

Feb'y 17. Found him moribund. Learned that he had been quite delirous until one o'clock this morning, since which he had slept considerably at intervals. He had taken his wine and broth at the usual breakfast hour, and the nurse did not then notice anything immediately alarming. He was soon after called by the patient, who complained of feeling "very curious," and then sank away so rapidly that in a very few minutes he was completely insensible,and was expiring by the time I reached him, although but a short distance from him; and summoned immediately.

The treatment consisted, at first, in the use of the neutral mixture, spts. nitre, and small doses of the blue mass with Dov. powder. The last two days,wine-whey, brandy and chicken-broth were chiefly relied upon.

Post-mortem, fifty hours after death. The body but slightly emaciated, abdomen more swollen than before death. The spleen was twice its natural size, being six inches long, three inches wide, with a corresponding increase in thickness, its tissue very soft and easily lacerated. The mesenteric glands enlarged to the size of Madeira nuts,and inflamed. The stomach had a large patch at the fundus highly injected, and in two places the coats were much thinned ; effusion of air under portions of the mucous coat. This effusion or collection of air was also noticed in the sub-peritoneal coat at the root of the mesentery, and in the sub-mucous tissue of portions of the small intestine. The duodenum was moderately injected, the jejunum pallid. At the upper portion of the ileum were noticed the isolated follicles much enlarged and inflamed at their base, their summits not ulcerated. Lower in the intestine these became more and more numerous, and also ulcerated at their summits; otherwise presenting the same characters as those first noticed. At the lower portion of the ileum there were from *twelve* to *eighteen* of these enlarged glands of Brunner to the square inch, and covering thus thickly the whole of that part of the intestine.

Not less than *thirty-two* of the elliptical glands of Peyer were counted in the ileum, none less than half an inch by three-quarters. The largest one was two inches in length, by one and a quarter inches in width. The major portion of these were in the lower *four feet* of the ilium. There were many smaller ones not enumerated, scattered through the ileum. These glands were all elevated, ulcerated, thickened, presenting the honey-combed appearance in every instance, with a dirty greyish slough or secretion in the interstices of tbe bands which crossed them and each other. The edges of some of them were everted. None had reached the point of perforation. The mucous membrane of the lower half of the ileum was generally of a slate color, without any bright red, or inflammatory injection.

The caput-coli was one mass of enlarged and inflamed follicles. These enlarged follicles were also thickly scattered all through the colon, but becoming *very gradually* less numerous as we approached the rectum. The mucous membrane was generally removed from their top by ulceration. The general tinge of the mucous membrane of the colon was a bluish-brown.

This was a most interesting case of fever. The young man, laboring under long continued mental depression, was taken with a light chill, which recurred every afternoon for twelve days before he gave up to it and went to bed. The second day after this (the fourteenth of the fever) he was brought to the Hospital, without any prominent symptoms, except his continued fever, some thirst, general restlessness, and some annoying, wandering pains. He remained in this state for two days, when his pulse began to rise, the cerebral symptoms became more marked and, continued to increase in severity for two days, when he died.

The latency of the enormous amount of disease of the bowels is somewhat remarkable, although the same thing has been often before remarked and recorded. The regularity of the return of the chills for twelve days is particularly worth noting. Had he been under the care of any physician, quinine would undoubtedly have been prescribed, but with what effect must of course remain uncertain. This, too, would naturally lead one to suppose he was laboring under a low form of remittent fever, and thus I supposed for the first thirty-six or forty-eight hours after his entrance, until, disappointed in their expected return, with an entire absence of yellowness of the skin, and the evident affection of the brain which became more marked at this period, led me to a correct conclusion as to his disease two days before he died. The autopsy will show the entire inadequacy of any course of treatment that we possess or might have adopted, to the accomplishment of his recovery. Yours truly,

G. N. BURWELL.

